# *In vitro* Label Free Raman Microspectroscopic Analysis to Monitor the Uptake, Fate and Impacts of Nanoparticle Based Materials

**DOI:** 10.3389/fbioe.2020.544311

**Published:** 2020-10-29

**Authors:** Hugh J. Byrne, Franck Bonnier, Esen Efeoglu, Caroline Moore, Jennifer McIntyre

**Affiliations:** ^1^FOCAS Research Institute, Technological University Dublin, Dublin, Ireland; ^2^UFR Sciences Pharmaceutiques, EA 6295 Nanomédicaments et Nanosondes, Université de Tours, Tours, France

**Keywords:** Raman microspectroscopy, nanoparticles, *in vitro* cytotoxicity, polystyrene nanoparticles, poly(amidoamine) dendrimers, molybdenum disulphide nano plates, high content spectroscopic analysis

## Abstract

The continued emergence of nanoscale materials for nanoparticle-based therapy, sensing and imaging, as well as their more general adoption in a broad range of industrial applications, has placed increasing demands on the ability to assess their interactions and impacts at a cellular and subcellular level, both in terms of potentially beneficial and detrimental effects. Notably, however, many such materials have been shown to interfere with conventional *in vitro* cellular assays that record only a single colorimetric end-point, challenging the ability to rapidly screen cytological responses. As an alternative, Raman microspectroscopy can spatially profile the biochemical content of cells, and any changes to it as a result of exogenous agents, such as toxicants or therapeutic agents, in a label free manner. In the confocal mode, analysis can be performed at a subcellular level. The technique has been employed to confirm the cellular uptake and subcellular localization of polystyrene nanoparticles (PSNPs), graphene and molybdenum disulfide micro/nano plates (MoS_2_), based on their respective characteristic spectroscopic signatures. In the case of PSNPs it was further employed to identify their local subcellular environment in endosomes, lysosomes and endoplasmic reticulum, while for MoS_2_ particles, it was employed to monitor subcellular degradation as a function of time. For amine functionalized PSNPs, the potential of Raman microspectroscopy to quantitatively characterize the dose and time dependent toxic responses has been explored, in a number of cell lines. Comparing the responses to those of poly (amidoamine) nanoscale polymeric dendrimers, differentiation of apoptotic and necrotic pathways based on the cellular spectroscopic responses was demonstrated. Drawing in particular from the experience of the authors, this paper details the progress to date in the development of applications of Raman microspectroscopy for *in vitro*, label free analysis of the uptake, fate and impacts of nanoparticle based materials, *in vitro*, and the prospects for the development of a routine, label free high content spectroscopic analysis technique.

## Introduction

Nanoscience and technology are novel, rapidly emerging fields which encompass the design, production, and exploitation of novel structures, processes and devices at the scale of 1–100 nm. Numerous applications based on nanoparticles have already been marketed, in products as diverse as industrial lubricants, advanced tires, self-cleaning glass, paints, semiconductor devices, medicines, cosmetics, sunscreens, nutraceuticals and food (Nano tech project, 2020). The international nanomaterials market was estimated to be $14,741.6 million in 2015, and is expected to increase to $55,016 million by 2022 (Allied market research, 2020). Specifically in the field of biomedicine, by 2015, nanotechnology had already created a 96.9 billion $US market (Pattni et al., 2015) and is projected to continue to have a significant impact. Polymeric micro and nanoparticles (NPs) have been explored for a wide range of medical applications in diagnosis, tissue engineering, and as drug delivery vehicles (Storrie and Mooney, 2006; Ito et al., 2008; Naha et al., 2008, 2009; Gao et al., 2016; Thaxton et al., 2016). Understanding the interface of these functional biomaterials with living systems and tailoring their capacity to target and penetrate cells is a short term challenge, which, if met, could revolutionize targeted drug and nutrient delivery, as well as other therapeutic strategies. On the other hand, major concerns have been raised regarding the possible human and environmental effects of accidental, large scale, NP exposure, in the short and long term. Profiling the interactions of nano particulate materials with human cells, their update and fate, as well as the cellular responses induced, is therefore of paramount importance (Love et al., 2012; Anguissola et al., 2014; Ajdary et al., 2018).

Among the main challenges is to detect and identify nanoparticles that have traversed the cell membrane, and to monitor their trafficking and fate within the call. One of the most commonly employed methods to do so, is that of confocal fluorescence microscopy, applied to imaging cells exposed to fluorescently labeled NPs *in vitro* (Roy et al., 2005; Cang et al., 2007; Naha et al., 2010a). Notably, however, not all NPs can be readily fluorescently labeled, and furthermore, it has been demonstrated that the label can be released into the surrounding cellular environment, such that the fluorescence distribution within the cell does not necessarily represent the presence or spatial distribution of the NPs (Suh et al., 1998; Yin Win and Feng, 2005; Salvati et al., 2011). It is also unclear whether the transport of NPs, particularly smaller ones, fluorescently labeled with anionic groups, is the same as that of their unlabeled counterparts. As an example, extrinsic labeling of bovine serum albumin with fluorescein-5-isothiocyanate has been reported to change its adsorption and diffusion behavior (Gajraj and Ofoli, 2000). Transmission Electron Microscopy (TEM) is also commonly used to visualize NPs and their surroundings within cells, but a considerable amount of sample processing (fixing and ultramicrotoming) is required, and, in order to visualize the NPs, they must have sufficient electronic contrast to the local environment of the cell (Davoren et al., 2007; Shapero et al., 2011). Ideally, techniques to localize and identify NPs internalized in cells, should be based on their intrinsic chemical composition, rather than electronic contrast or the properties of extrinsic labels. Identification of the local subcellular environment of NPs in the cytoplasm (e.g., endosomes, lysosomes, endoplasmic reticulum) or nucleus could further advance the understanding of their intracellular trafficking and interaction mechanisms, and the resultant impact on the metabolism of the cell. Such impacts are commonly and routinely screened *in vitro* by conventional cytotoxicity assays, such as the colourometric dye- based assays Alamar Blue, Neutral Red, MTT, etc, which can provide indications of impact on cell viability, proliferative capacity, metabolic activity, endosomal/lysosomal and mitochondrial activity (Mukherjee et al., 2010b). However, each assay reports on a single endpoint, such that multiple assays are required, at multiple time points, and doses. Furthermore, there have been numerous reports of false positive results of such assays, due to the extracellular interaction of the NPs with the *in vitro* cell culture medium (Casey et al., 2007a, 2008), and with the molecular constituents of the colorometric cytotoxicity assays themselves (Casey et al., 2007b), highlighting the need for alternative methodologies (Herzog et al., 2007).

As an alternative technique, label free Raman microspectroscopy can provide a holistic, real-time representation of the biochemistry of the whole cell, at subcellular levels, and has previously been employed for characterization of the biochemical evolution underpinning cell culture and mitosis (Boydston-White et al., 2006; Matthäus et al., 2006), as well as cell proliferation (Short et al., 2005), differentiation and activation (Notingher et al., 2004; Ami et al., 2008; Pavillon et al., 2018), cellular adhesion (Meade et al., 2007), death (Gasparri and Muzio, 2003), and invasion (Liu et al., 2001).

The Raman effect was first described in Raman and Krishnan (1928) and the principles and underlying theory are described in numerous excellent textbooks, notably that of Long (2002). Based on inelastic scattering of light due to coupling of energy of incident photons with vibrations of target samples, Raman spectroscopy is well established for chemically fingerprinting materials, with applications in, for example, forensics and the pharmacological industry (Hodges and Akhavan, 1990; Kneipp et al., 1999; Vankeirsbilck et al., 2002; Edwards and Vandenabeele, 2016). Modern instruments typically couples a laser to a microscope, which focuses the source light and also collects the scattered radiation. The sample can be rastered to produce a map of the sample, in a similar way to a laser scanning fluorescence microscope. Raman microspectroscopy can also be performed at UV, visible, or near IR wavelengths in a confocal mode and can provide detail at sub micrometer resolution. In this context, instrumentally, Raman microspectroscopy is similar to Confocal Laser Scanning Microscopy, except that the chemical specificity derives from the analysis of the spectrum of the scattered light, dispersed by a diffraction element (e.g., grating). Similar to infrared vibrational spectroscopic analysis and imaging, the technique therefore has the advantage of being truly label-free, producing a spectrum which comprises contributions from each molecular bond, and is a “signature” or “fingerprint” which is characteristic of a material, or changes associated with a physical or chemical process. In complex samples, notably biological cells or tissue, the spectroscopic signature incorporates characteristics of all constituent functional groups of lipids carbohydrates, proteins and nucleic acids (Byrne et al., 2010). A comparison of the techniques of Infrared and Raman spectroscopic imaging for biomedical applications is provided in Byrne et al. (2010). Notably, however, the Raman effect, is intrinsically weak, requiring the highly sensitive detection of Charge Coupled Detector arrays, and the features of the spectrum can often be swamped by fluorescence or stray light scattering (Byrne et al., 2010). Depending on the nature of the sample, and the desired signal to noise ratio, a single spectrum is typically accumulated over a period of seconds, and therefore a spatial map of a sample can take minutes or hours, depending on the desired area to be mapped, and sampling step size. Although time resolved Raman spectroscopy using pulsed laser sources can be utilized to study reaction mechanisms and correlations between molecular structure and reaction rates (Sahoo et al., 2011), commercial instruments typically use steady state conditions, but can measure the temporal evolution of a system by measuring at specific time points. More sophisticated variants, such as surface enhanced Raman scattering, and coherent Raman scattering can enhance the sensitivity of the technique, and will be discussed in more detail in section “In vitro toxicity assessment using Raman Microspectroscopy.”

Raman microspectroscopy has attracted increasing attention for biological characterization and biomedical applications, and specific protocols have been described (Butler et al., 2016). *In vitro* cellular analysis can be performed at subcellular level, of fixed or live cells, in 2D and/or 3D culture environments (Meade et al., 2010; Bonnier et al., 2011; Gargotti et al., 2018). Screening of multiple cell lines has demonstrated a remarkable reproducibility of the subcellular signatures, elucidating, for example, the role of the nucleolus in discriminating different cancer cell lines (Farhane et al., 2015b). The reproducibility of the signatures was seen to also extend to the comprehensive and systematic analysis of drug uptake and mechanisms of action (Farhane et al., 2015a, 2017a,b, 2018a,b; Szafraniec et al., 2016), radiotherapy (Meade et al., 2016; Roman et al., 2019) and nanoparticle toxicity (Dorney et al., 2012; Efeoglu et al., 2015, 2016, 2017a,b, 2018), demonstrating potential applications for high content analysis for pre-clinical drug screening and toxicological applications (Byrne et al., 2018).

Raman microspectroscopy potentially offers a label free, high content probe of NPs within cells, which can potentially analyze their local environment, their fate, and ultimately changes in the cellular metabolism which can be correlated with cytotoxic responses, oxidative stress, or inflammation. Illustrated with specific examples from the published work of the authors, this paper will present the principles and methodology of cellular and subcellular analysis using Raman microspectroscopy, and will detail some examples of multivariate chemometric data mining to identify the uptake and localization of NPs within cells, and to explore the spectroscopically fingerprint of the local environment of trafficking, or degradation, over time. The dose and time dependent evolution of spectroscopic signatures, and their biochemical origin, will be described, and correlated with conventional cytoxicological approaches. As well as mechanisms of acute cytotoxicological responses, genotoxic responses will also be explored.

## Raman Microspectroscopic Profiling of Cells

Raman microspectroscopic profiling of biological cells, *in vitro*, is typically performed by point mapping over a grid defined within the field of view of the objective. The cells can be profiled live, in the culture medium, although the presence of the dye phenol red can obscure the visual image, and so mapping in saline solution or phenol red free medium is often performed (Bonnier et al., 2010a). Measurement in immersion minimizes background scatter (Bonnier et al., 2011) and any photo-thermal degradation (Bonnier et al., 2012), and substrate contributions to the spectra can be avoided by culturing on 3D protein matrices such as collagen, such that high signal to noise/background spectra can be obtained, the background being the spectrum of water/medium (Bonnier et al., 2010b).

**FIGURE 1:**
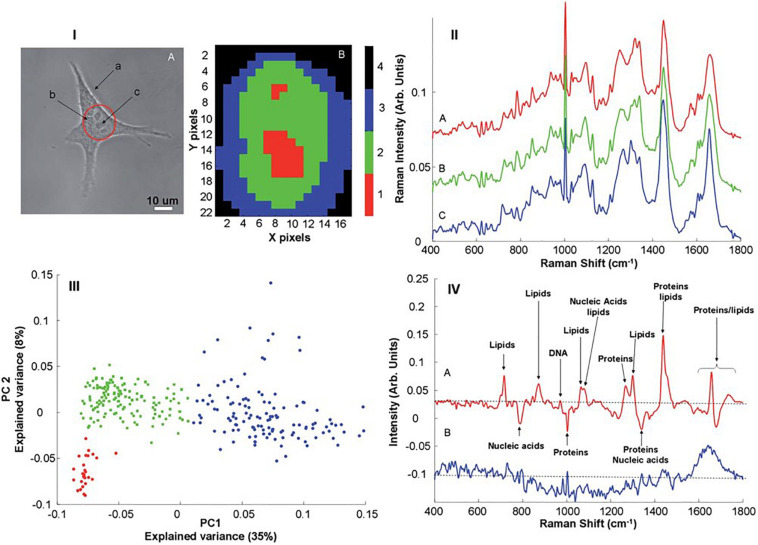
**(I)** (A) Typical bright field image of an A549 cell. The different structures such as membrane (a), cytoplasm (b) and nucleus (c) are clearly identifiable. The nucleolus present inside the nucleus can also be seen. The area delineated by red indicates a “typical area” selected for Raman mapping (B), Example of K-means reconstructed image from a Raman map recorded on the nuclear area of an A549. In both X and Y directions, 1 pixel corresponds to a mapping step of 1 mm. **(II)** Mean spectrum calculated for the different clusters obtained after K-means clustering analysis corresponding to the nucleoli (A), nucleus (B) and cytoplasm (C). **(III)** Scores plot of the first two principal components after PCA performed on Raman spectra recorded from A549 cells. The individual data points have been color coded according to the results of K-means cluster analysis; nucleus (green), nucleolus (red) and cytoplasm (blue). **(IV)** Plot of the loadings of PC1 (A) and PC2 (B). Different features corresponding to the lipids, proteins and nucleic acids can be identified (Bonnier and Byrne, 2012).

Figure 1 shows examples of Raman microspectroscopic maps of an individual A549 human lung adenocarcinoma cell (Bonnier and Byrne, 2012). In the bright field image of Figure 1(IA), the cell cytoplasm and nucleus are clearly visible, and nucleoli within the nuclear region can be identified. Figure 1(IB) shows a false color image of the same cell, after KMCA of the Raman spectroscopic data. A high degree of correspondence between the distribution of the clusters and the substructure of the cell can be clearly seen, and the nucleoli, nucleus and cytoplasm are associated with individual clusters. The spatial definition of the map is determined by the objective employed, and the mapping step size. The use of a 100x objective provides the maximum lateral resolution (∼1 mm) and provides visible images which elucidate the cell morphology. Decreasing the lateral step size from 1.5 to 0.75 mm was seen to improve the definition of the subcellular structures (Dorney et al., 2012). Uzunbajakava et al. (2003) have reported that, using intervals 2–3 times smaller than the laser spot, optimal lateral sampling can be achieved, promising even higher definition of the subcellular features.

K-Means Clustering Analysis (KMCA) false color maps give a representation of the biochemical variability across the cell, each cluster representing regions of similar biochemical character. The biochemical information of each cluster is represented by its mean spectrum, in which characteristic bands of proteins, lipids, nucleic acids and carbohydrates can be identified. Figure 1(II) shows the mean spectra obtained from selected clusters of the KMCA image of Figure 1(IB) [nucleoli (A), nucleus (B), and cytoplasm (C)]. The characterization of live cells has been reported in a number of studies (Notingher et al., 2003; Short et al., 2005; Notingher and Hench, 2006; Swain et al., 2008; Bonnier et al., 2010a) and, assignments of the different peaks can be made, and are well cataloged in literature (Movasaghi et al., 2007). Differences between the mean spectra are subtle and not easily discernible, however, but can be better analyzed using Principal Components Analysis (PCA), by which the spectra of each individual cluster can be compared. Figure 1(III) shows a PCA scatter plot (PC 1 versus PC 2) for the spectra of the clusters corresponding to the nucleus (green), nucleolus (red) and cytoplasm (blue). PC 1, representing 35% of the explained variance, indicates good discrimination between the spectra of the cytoplasm and those of the nuclear regions. The spectra of the two regions of the nucleus are partially differentiated by PC2, but can be more clearly discriminated in a pairwise analysis (Bonnier and Byrne, 2012).

The spectral loadings represent the variability described by a given PC, as a function of wavenumber, and are used to identify the key spectroscopic features which differentiate the respective clusters, which can in turn by associated with specific biochemical constituents of the cell. Figure 1(IVA) shows the loading of PC1, which enables visualization of the spectral features responsible for discrimination between the cytoplasm and the nuclear regions. Positive contributions to the loading are associated with spectra which score positively according to the PC in the scatter plot, and vice versa for negative peaks (Bonnier and Byrne, 2012), and thus the features of the loading can be analyzed in terms of reference spectra of known biomolecules. The prominence of, for example, DNA and RNA in the nucleus of the cell is clear, while lipidic features are stronger in the cytoplasm of the cell. It has been demonstrated that a pairwise PCA of datasets better facilitates the interpretation of the loadings, however, and enables differentiation of the subcellular nuclear regions based on nucleic acid content (Bonnier and Byrne, 2012).

Raman microspectroscopy, coupled with multivariate chemometric analysis, is clearly therefore a powerful, label free methodology for high content analysis of cells, with optical spatial resolution. However, a single cellular map can take anything between minutes to hours to complete (Bonnier et al., 2010a; Bonnier Knief et al., 2011), depending on the laser spot, step size and desired quality of signal, and, as a result, only a small proportion of the cell population is typically analyzed (Butler et al., 2016). In order to produce datasets of a larger cell population, a “point spectra” approach is commonly adopted, whereby the subcellular regions of nucleolus, nucleus and cytoplasm of multiple cells in a cell culture are sampled, and subsequently data mined using multivariate analysis. Erring on the side of prolonged acquisition times for high quality spectral analysis, the cells are typically formalin fixed and air dried (Hobro and Smith, 2017). This methodology has been employed, for example, to understand the subcellular differentiation of lung cancer (Farhane et al., 2015b) and oral cancer cell lines (Carvalho et al., 2015), and to monitor the subcellular accumulation of, binding interactions, and subsequent cellular response to chemotherapeutic agents (Farhane et al., 2015a, 2017a,b, 2018c).

## Localization and Trafficking of Nanoparticles in Cells

**FIGURE 2:**
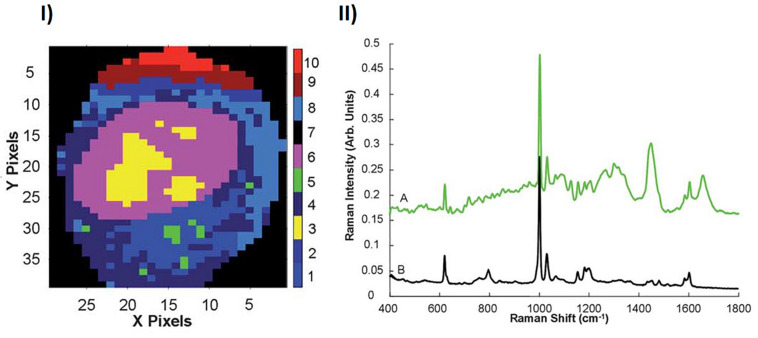
**(I)** KMCA map of the Raman profile of the nuclear and perinuclear area of an A549 cell. **(II)** KMCA spectrum of Cluster 5 (A), compared to the Raman spectrum PSNPs (B). Spectra are offset for clarity (Dorney et al., 2012).

The analysis protocols of Section 2 were employed to similarly profile A549 cells exposed to non-toxic polystyrene nanoparticles (PSNPs). In addition to clusters of the nucleoli (cluster 3), nucleus (cluster 6) and cytoplasm (clusters 1,2,4,8,9,10), KMCA identifies a spectral cluster (cluster 5) which has strong features which are characteristic of polystyrene, as shown in Figure 2. The spectral features of this cluster unambiguously confirm the localization of the NPs within the cytoplasmic and perinuclear region of the cell (Dorney et al., 2012). Notably, the mean spectrum of cluster 5 clearly also contains biological contributions, and so the analysis can potentially provide information about the local cellular environment of the NPs, and the *in vitro*, intra cellular trafficking process.

**FIGURE 3:**
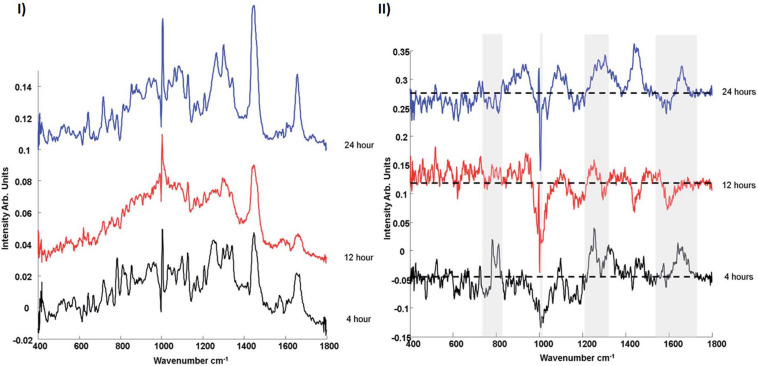
**(I)** Comparison of mean spectra of 4, 12, and 24 h nanoparticle exposure data sets. **(II)** Comparison of PCA loadings of 4, 12, and 24 h nanoparticle exposure data sets. Spectra are offset for clarity. The dotted line represents the ‘0’ line for each loading (Efeoglu et al., 2015).

Efeoglu et al. (2015) using confocal laser scanning fluorescence microscopy and organelle staining, analyzed the uptake and localization of similar non-toxic carboxylated PSNPs in A549 cells, as a function of exposure time, confirming colocalization in endosomes (4 h), lysosomes (8 h), and endoplasmic reticulum (24 h). In parallel, Raman microspectroscopic profiling of multiple cells in a Raman mapping approach, combined with KMCA and PCA, was used to differentiate the local biochemical environment of the NPs at the different timepoints, and identify the spectroscopic signatures of the associated subcellular organelles (Figure 3). The contribution of the spectrum of the PSNPs themselves was calculated using a Least Squares analysis, and subtracted (Efeoglu et al., 2015).

PSNPs are initially endocytosed by cells (Salvati et al., 2011), whereupon they are bound by membrane derived endosomal vesicles. After 4 h particle exposure, compared to spectra of 12 and 24 h particle exposure, the Raman spectrum is seen to exhibit strong bands attributed to proteins, within the range of 700–900 cm^–1^, which exist naturally in membrane structures (Terasaki and Jaffe, 1991; Notingher and Hench, 2006; Talari et al., 2015). Bands related to phospholipids (∼1080, ∼1656 cm^–1^) and phosphatidylcholine and membrane lipids (∼790, ∼718 cm^–1^) are consistent with membrane derived vesicles. Early endosomes are internally trafficked within the cytoplasm and are engulfed by lysosomes, which originate from the golgi apparatus and endoplasmic reticulum, and therefore show similar properties (Watson et al., 2005). The Raman signature which characterizes the 12 h exposure data set is similarly dominated by features of proteins and lipids, although the their signatures are significantly different to those which characterize the early endosomes. Prominent bands related to phosphatidylinositol (519 cm^–1^), phosphatidylethanolamine (759 cm^–1^), C–C stretch of lipids (1130 cm^–1^), CH_2_ twist of lipids (1304 cm^–1^) and CH_def_ of lipids (1439 cm^–1^) are observed. Compared to the 4 h, endosomal spectrum, the protein derived features in the range 700–900 cm^–1^ disappear, whereas clear, protein related differences can now be observed in the amide I (∼1600–1700 cm^–1^) and amide III (∼1200–1300 cm^–1^) regions. PSNPs containing lysosomes are trafficked to the golgi apparatus or endoplasmic reticulum (Chang et al., 2008; Dorney et al., 2012). After 24 h, the mean spectrum, associated with the endoplasmic reticulum, is characterized by prominent bands of nucleic acids (∼785 cm^–1^ and 810 cm^–1^), consistent with the presence of RNA required for protein synthesis within the perinuclear region and granular endoplasmic reticulum. Generally, the characteristic spectral profile is consistent with the protein lipid and rich nature of the endoplasmic reticulum.

Other studies have demonstrated the use of Raman imaging to visualize the uptake of nanoparticles, such as metallacarborane aggregates into single cells (Schwarze et al., 2020), and magnetic nanoparticles into erythrocytes (Soler et al., 2007). Carotene, an extremely strong Raman scatterer, was employed as a surface coating of carbon nanoparticles, such that their uptake could be readily visualized using Raman mapping of human melanoma and breast cancer cells, *in vitro* (Misra et al., 2016). Chernenko et al. (2009) also employed Raman microspectroscopy to monitor the intracellular delivery of two biodegradable nanoparticle systems, commonly used as drug delivery vehicles, poly(ε-caprolactone) and poly(lactic-co-glycolic acid), and their subcellular degradation patterns. The nanoparticles were identified in the subcellular environment of lysosomes, and the degradation pathway was mapped by shifts and intensity changes of the characteristic spectral profiles of the materials.

**FIGURE 4:**
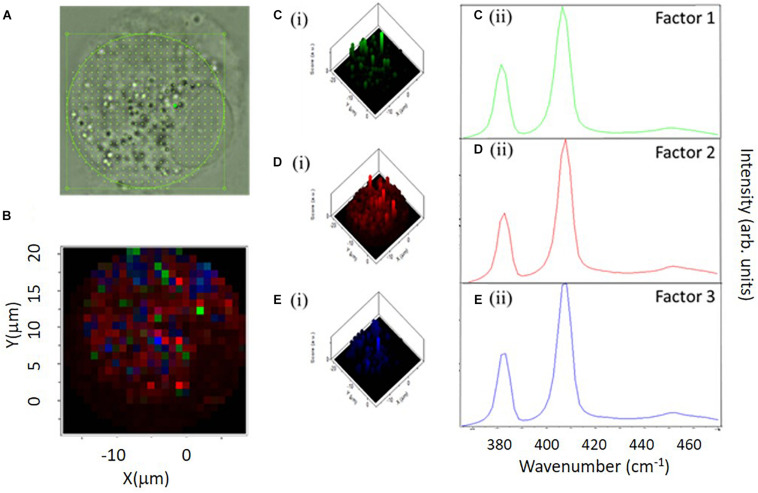
Raman Map Analysis of a macrophage-like THP-1 cell following a 24 h incubation. Spectral range 365–470 cm^–1^. **(A)** Bright field image of THP-1 cell under x100 water immersion and taken on a Horiba dual Raman microscope. **(B)** Figure 2D overlay image of the Raman map showing the location of the three Factors (Factor 1 – green, Factor 2 – red, Factor 3– blue). **(C)** (i) 3D construction showing the location of Factor 1 within the cell. **(C)** (ii) Mean spectra of Factor 1 displayed. **(D)** (i) 3D construction showing the location of Factor 2 within the cell. **(D)** (ii) Mean spectra of Factor 2 displayed. **(E)** (i) 3D construction showing the location of Factor 3 within the cell. **(E)** (ii) Mean spectra of Factor 3 displayed (Moore et al., 2020a).

Label-free Raman micro-spectroscopy was similarly employed to confirm the intracellular localization and fate of molybdenum disulfide (MoS_2_) submicron plates, in differentiated THP-1 macrophage like cells, *in vitro* (Moore et al., 2020a). The field of 2D materials technology has significantly expanded since the isolation and characterization of monolayer graphene in 2004 (Novoselov et al., 2004) and the emergence of techniques for liquid phase exfoliation (Smith et al., 2011). It is critical, at this juncture, to understand how such materials interact with cells following exposure, and the influence of the cellular micro-environment on the physico-chemical properties of these particles. Macrophages play a crucial role in recognizing a foreign threat, causing a cascade of events designed to eliminate them from their environment and to maintain homeostasis (Gordon, 2007). Using sub-lethal doses (Moore et al., 2017), internalized MoS_2_ submicron plates were clearly identifiable in THP-1 cells by their characteristic Raman spectroscopic features, the E^1^_2__g_ (380 cm^–1^) and A^1^_g_ (407 cm^–1^) peaks (Moore et al., 2020a). Using a combination of single cell mapping and multicellular point spectral Raman microspectroscopic analysis, three distinct local environments of the particulate material were identified in untreated THP-1 cells, and 4, 24, and 72 h after a 4 h exposure to MoS_2_ submicron plates, in a pulse chase approach. In all cases, white light microscopic images revealed that the cells were rich in vesicles of serval microns diameter particularly in the perinuclear region (Figure 4A). Analysis of the spectra of the prominent vesicles in multiple cells, in a point spectra approach, indicted that those of untreated macrophage cultures were characterized by spectroscopic features of sphingomyelin lipids, normally prominent in the cell membrane, and are therefore associated with the transport of the membrane lipid from the endoplasmic reticulum. In contrast, those of cells 4 and 24 h after exposure to MoS_2_ were predominantly characterized by signatures of phosphatidyl lipids, and are therefore associated with phagosomes and/or phagolysosomes. Seventy two hours after exposure the population of intracellular lipidic vesicles was again dominated by those of a sphingomyelin character, indicating a return to homeostasis (Moore et al., 2020b). Mapping individual cells, 4, 24, and 72 h after exposure, in addition to vesicles with prominent features of phosphatidyl lipids, another species of vesicle, with strong signatures of lysozyme, was identified (Moore et al., 2020a). Using Factor Analysis (Figure 4B), different spectral signatures of MoS_2_ were identified (Figures 4Cii,Dii,Eii) distributed differently throughout the cell (Figures 4Ci,Di,Ei). The spectral profiles of the second two factors (Figures 4Dii,Eii) are consistent with those of degraded MoS2, using PCA, were associated with the phosphatidyl environment, after 24 h, but not in the lysosymal, suggesting that the activity of the enzyme is inhibited by the lipopolysaccharide (LPS) contaminant, previously identified on the surface of particulate material (Moore et al., 2017).

## *In vitro* Toxicity Assessment Using Raman Microspectroscopy

The potential of Raman microspectroscopy to probe the *in vitro* cytotoxicity to nanoparticle exposure was first explored by Knief et al. (2009), for the example of exposure of A549 cells to single walled carbon nanotubes. Peak ratio analysis of modes of lipidic CH_2_ deformation (∼1302 cm^–1^) as well as DNA bases guanine, adenine and thymine (∼1287 and ∼1338 cm^–1^) versus the amide III band (∼1238 cm^–1^), demonstrated a dose dependent response which correlated with previous toxicological studies (Davoren et al., 2007). Notably, these modes had previously been employed in a study of HgCl_2_ toxicity to human keratinocytes, *in vitro* (Perna et al., 2007). PCA was employed to elucidate the dose dependent cellular response and associated characteristic spectroscopic signatures. To further illustrate the potential of Raman microspectroscopy in this field, partial least squares regression analysis combined with genetic algorithm feature selection were employed to demonstrate that the end points of the clonogenic assay (Herzog et al., 2007), as a measure of the toxic response, can be predicted from the Raman spectra of cells exposed to undetermined doses, potentially eliminating the requirement for time consuming and costly cytotoxicological assays. However, similar studies demonstrated that there was no evidence that the nanotubes were internalized in the cells (Davoren et al., 2007), and that an indirect toxicity, due to medium depletion (Casey et al., 2007a) was the most likely cause of the cellular response.

PSNPs serve as a model for NP uptake in cells (Anguissola et al., 2014), and can be fluorescently labeled for tracking and organelle co-localization studies (Salvati et al., 2011; Efeoglu et al., 2015). While their carboxylated counterparts are non-toxic, amine functionalized PSNPs (PS-NH_2_) elicit a well documented, dose and time dependent *in vitro* toxic response, associated with oxidative stress, and inflammatory cascades, leading to apoptosis (Anguissola et al., 2014; Maher et al., 2014). Using the protocol of point spectral analysis (cytoplasm, nucleus, nucleoli) of control and exposed cells, the dose and time dependent responses have also been monitored using Raman microspectroscopy, demonstrating the potential of the technique as a label free high content screening protocol (Efeoglu et al., 2016).

**FIGURE 5:**
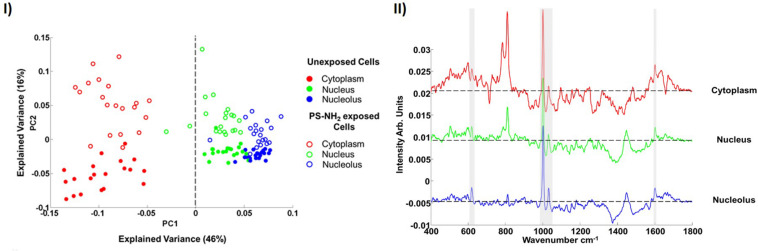
**(I)** Scatter plot of the PCA of spectra corresponding to cytoplasm, nucleus and nucleolus of the (8 h) unexposed and PS-NH_2_ exposed cells for 10 μM concentration of nanoparticles. Different cellular regions are coded as follows; red for cytoplasm, green for nucleus and blue for nucleolus. Exposed and unexposed cells are indicated by open circles and closed circles, respectively. **(II)** Mean difference spectra of cytoplasm (red), nucleus (green) and nucleolus (blue) obtained by subtraction of mean spectra of 8 h PS-NH_2_ exposed cells from mean spectra of unexposed cells. The spectra are offset for clarity, the dashed line indicating the zero point. The bands related to PS are indicated with gray highlights (Efeoglu et al., 2016).

After an 8 h exposure to a 10 μM concentration of nanoparticles, the spectra of each subcellular region of the cells can be differentiated, using PCA, from those of the control cells, as shown Figure 5I. Although subcellular vesicles are not specifically targeted, the mean difference spectra (Figure 5I) clearly show features of the PS-NH_2_, which are excluded from the analysis. The PCA loading (Figure 5II) also show clear biological signatures, particularly in the cytoplasmic region, which can be associated with the cytotoxic response, and ultimately correlated with conventional *in vitro* cytotoxicity assays.

**FIGURE 6:**
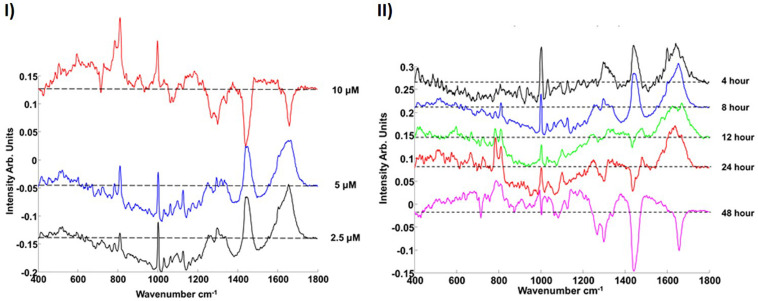
**(I)** Loadings of PC1 for pairwise analysis of cytoplasm of PS-NH_2_ exposed cells with the control for 10 μM (red), 5 μM (blue), and 2.5 μM (black) after 8 h exposure. **(II)** Comparison of the Loading of PC1s for different (2.5 μM) PS-NH_2_ exposure times (cytoplasm). 4, 8, 12, 24, and 48 h are indicated with black, blue, green, red, and magenta, respectively. The dotted line represents the zero ‘0’ point for each loading. Loadings are offset for clarity. Positive features of the PCs are related to exposed cells while negative features of the PCs are related to their controls (Efeoglu et al., 2016).

In the dose and time dependent responses of A549 cells to PS-NH_2_ exposure, the most prominent spectral marker which differentiates the cytoplasm of the control and exposed cells is seen to be the a ‘doublet’ of peaks at 785 and 810 cm^–1^ (Figure 6). The appearance of this feature is indicative of changes to the cytoplasmic RNA content associated with oxidative stress, and is observed in cells even at low doses, and short exposure times (4 h). The intensity of the band is seen to systematically and progressively change as a function of both NP dose and exposure time (Efeoglu et al., 2016) and its evolution is associated with concurrent and subsequent changes in protein (Amide I region, ∼1600 – 1700 cm^–1^) and lipid (1229 and 1438 cm^–1^) content and/or structure, indicative of proteolysis and lipotoxicity (Efeoglu et al., 2016, 2017a).

**FIGURE 7:**
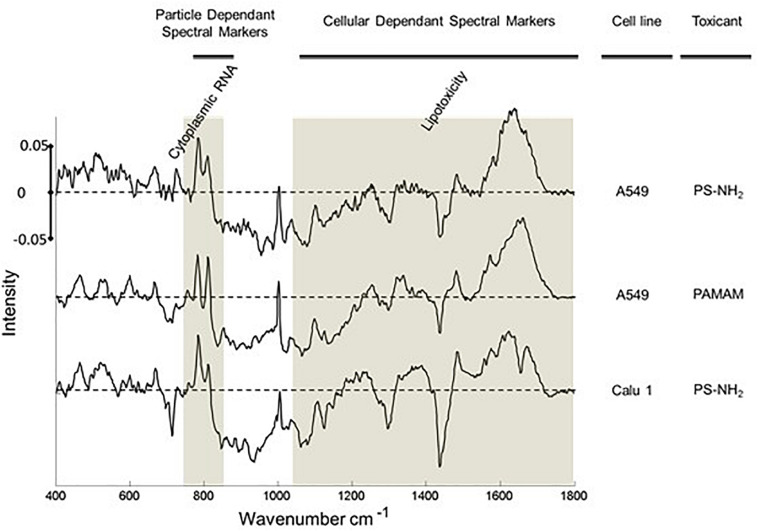
Spectral Markers of acute toxic response in the cytoplasm of A549 and Calu-1 (human lung epidermoid) cells after 24 h exposure to PS-NH_2_ and PAMAM nanoparticles. Positive and negative features of the loadings relate to exposed and unexposed cells, relatively. The 750–830 cm^– 1^ and above 1000 cm^– 1^ region are indicated with highlights. Loadings are offset for clarity. The dotted line represents the zero ‘0’ point for each loading and intensity scale of 0 ± 0.05 is used for comparison (Efeoglu et al., 2018).

The potential of Raman microspectroscopy to delivering multi-parametric information by label free analysis was further explored by comparing the evolution of spectral markers in other cancerous and non-cancerous cells lines (Efeoglu et al., 2017a). Characteristic spectral markers of toxic events, including oxidative stress and lysosomal damage, have been identified and characterized as a function of time, and the analysis indicates that the spectral markers identified for cellular dependent events are consistent across multiple cell lines (Figure 7), potentially facilitating the identification of the mechanism of toxic response to the nanomaterial. Analysis of the presence and progression of spectral markers, especially in the low wavenumber region, also indicates the applicability of Raman spectral analysis to identification of cellular signatures characteristic of cell death pathways in cancerous and non-cancerous cell lines, differentiating between necrotic and apoptotic (Efeoglu et al., 2017a, 2018).

Aminated dendritic polymer nanoparticles, such as poly (amidoamine) (PAMAM) and poly (propylene imine), have been demonstrated to elicit a similar profile of toxic responses in mammalian cells, *in vitro*, involving endocytosis, oxidative stress, mitochondrial damage, inflammatory cascades, leading to apoptosis (Mukherjee et al., 2010a,b; Naha et al., 2010b; Maher et al., 2014; Khalid et al., 2016). Notably, under similar exposure conditions, A549 cells were shown to exhibit remarkably similar (differential) spectroscopic profiles in the cytoplasm of A549 cells, associated with acute cytotoxicity (Figure 7) (Efeoglu et al., 2017a). Notably, the significantly smaller PAMAM dendrimers (∼5–7 nm) have been shown to elicit significant genotoxic responses *in vitro* (Naha and Byrne, 2013; Naha et al., 2018). In the case of A549 exposure to PAMAM dendrimers, a significant dose and time dependent response of the spectroscopic profile of the nuclear region was observed, which may be associated with DNA damage and a genotoxic response (Efeoglu et al., 2017a).

In a study of the intracellular uptake and toxic response of silver and copper oxide nanoparticles in human lung cells, *in vitro*, and Raman spectroscopy was employed to monitor the cellular uptake, compared to the respective metal ionic species as well as the particle-cell interactions, although specific signatures of cellular responses were not identified (Cronholm et al., 2020). Raman spectroscopic imaging has also been employed in conjunction with microinjection of nanoparticles into cells, as a proposed tool for nanotoxicological studies (Candeloro et al., 2011).

Raman spectroscopy has also been employed to confirm the *in vitro* cytotoxicity, and therefore anti-cancer activity, of functionalized silica nanoparticles (Dhinasekaran et al., 2020). The surface of the silica nanoparticles was functionalized with 5-Fluorouracil by direct conjugation or chitosan mediated conjugation. Raman spectroscopy indicated changes in the nucleic acid spectral signature for cancer cell lines exposed to the formulation, rather than normal cells confirming a selective toxicity to cancer cells.

In a study of the impact of nanoparticle uptake on the biophysical properties of cells, Raman spectroscopy was employed in particular to analyze disruptions to the cell membranes (Rasel et al., 2019). The study indicated that nanoparticle uptake results in substantial perturbation of the fundamental structure of the cells, having implications for applications in biomedical engineering.

## Discussion

Technological advances in recent decades have rendered confocal Raman microspectroscopy increasingly routine amongst benchtop laboratory techniques for materials and process analysis. As it can be performed using optical sources of wavelength from the UV to near IR, it can provide submicrometer spatial resolution and therefore can image cells and processes at a subcellular level, and therefore a wealth of information about their biomolecular makeup, and changes to it due to external factors. As a label-free technique, it can unambiguously identify the presence of NPs within cells, on the basis of their intrinsic spectroscopic fingerprint, and can also profile the biochemistry of the local environment, differentiating endosomal, lysosomal and perinuclear compartmentalization in the course of NP uptake and trafficking by the cell. The stability of the NP material within the subcellular environment can also be probed, enabling mapping of catabolic pathways. In terms of the cytotoxic responses of the cells, clear dose and time dependent signatures, associated with oxidative stress, proteolysis and lipotoxicity, can be observed in the cytoplasm, and the processes of apoptosis and necrosis can be differentiated. In the nuclear regions, signatures of genotoxicity are indicated. The single, label-free technique can therefore provide *in vitro* high content spectroscopic screening of the process of NP uptake, trafficking, fate and acute cytotoxic and genotoxic responses. The technique is therefore ideally suited to address many of the short-term and long-term challenges of exploring the interface of functional (nano) materials with living systems, at least in the context of *in vitro* models.

**FIGURE 8:**
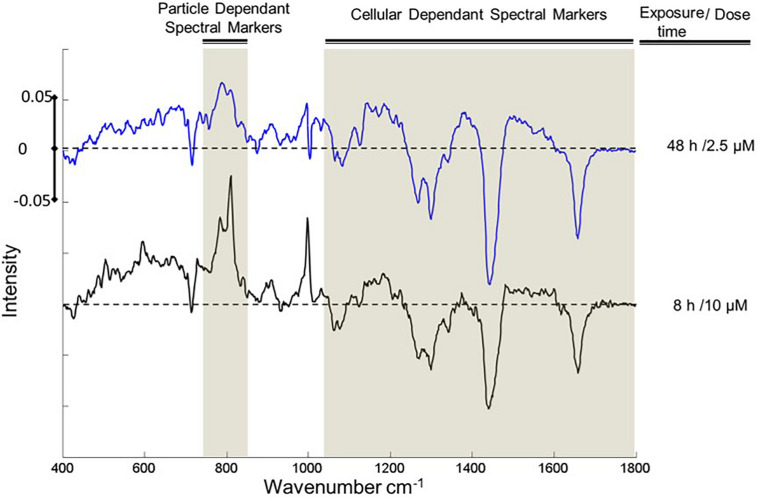
Comparisons of the loading 1 of PCA of cytoplasm for exposed and control cells, after 8 h exposure to 10 μM (red) and 48 h 2.5 μM (blue) PS-NH_2_. Positive and negative features of the loadings relate to exposed and control cells, respectively. The areas that show similar responses are indicated with highlights. Loadings are offset for clarity. The dotted line represents the zero ‘0’ point for each loading and intensity scale of 0 ± 005 is used for comparison.

**FIGURE 9:**
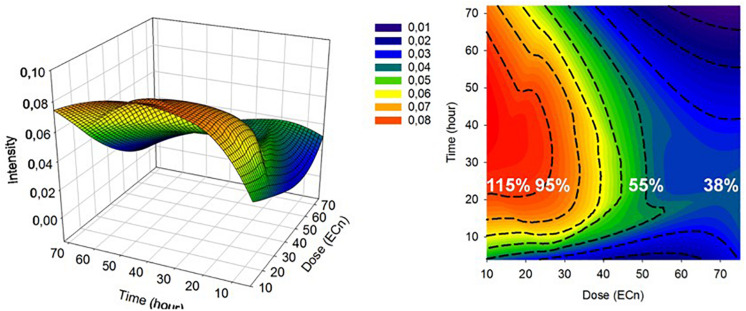
3D mesh and contour plot of 785 **(A,B)** spectral marker intensity as a function of time (h) and dose (EC_n_). The 24 h cellular viability at EC_10_, EC_25_, EC_50_, and EC_75_ determined by AB assay are indicated with percentages on the plot (white) (Efeoglu et al., 2018).

An important consideration is the reproducibility and transferability of the signatures of the spectral response, across NPs of similar cytotoxic mechanisms, and different cell lines. Previously, it has been demonstrated that the differential spectroscopic signatures (PC loadings) which discriminate the subcellular regions of cytoplasm, nucleus and nucleoli, of different cell lines are remarkably similar, so much so that different lung cancer cell lines could only be discriminated according to the signatures of the nucleoli (Farhane et al., 2015b). Furthermore, Figure 7 clearly indicates similar spectroscopic responses for two different aminated polymeric particles, both known to initiate oxidative stress responses, in two different cell lines. As shown in Figure 8, for the case of exposure of A549 cells to PS-NH_2_, the differential spectral profiles of low dose/prolonged exposure and high dose/short exposure also show remarkable similarities (Efeoglu et al., 2018). The 3D nature of the dose and time dependent spectral signatures is consistent with previous modeling analysis of dose and time dependences of *in vitro* cytotoxic responses (Maher et al., 2014; Byrne and Maher, 2019). The 3D contour map of the spectral responses can also be correlated with the response of classical colorometric cytotoxicity assays, as shown in Figure 9, for the example of the 785 cm^–1^ feature of the response of A549 cells to PS-NH_2_ exposure, and the dose response curve of AB at 24 h (Efeoglu et al., 2018).

The consistency of the responses indicates that the signatures of the multiparametric responses, composed of contributions of multiple biochemical constituents, are characteristic of the mode of action, supporting a “spectralomics” approach to high content spectroscopic analysis (Byrne et al., 2018; Efeoglu et al., 2018; Farhane et al., 2018c). In Figures 7, 8, the lower wavenumber responses (∼800 cm^–1^) are associated with oxidative stress, and so can be considered to be dependent on the initial NP dose and intrinsic toxicity (degree of amination). The higher wavenumber responses (1000–1800 cm^–1^) can be associated with the subsequent cascade of cellular responses. In the framework of the predictive toxicology approach of Adverse Outcome Pathways (OECD, 2017; Wittwehr et al., 2017), the characteristic “spectralome” of the initial response can be considered that of the Molecular Initiating Event. The subsequent adverse pathway of cell death is described by the respective characteristics of inflammatory responses and apoptosis or necrosis in the higher wavenumber region of the fingerprint.

Critical for realizing the potential of the technique is reliably data mining the evolution of the signatures which map out the temporal evolution, and spatial propagation, of the key events of the subcellular responses. While multivariate algorithms such as KMCA and PCA provide a detailed picture of the static state of the cellular system, compared to control, more sophisticated data mining techniques are required to monitor the kinetic evolution of signatures of cellular response pathways. Algorithms such as Evolving Factor Analysis (EFA) (Keller and Massart, 1991) and Multivariate Curve Resolution Alternating Least Squares (MCR-ALS) (Felten et al., 2015) have been developed to similarly monitor the evolution of chemical and photochemical reactions. Constraints based on kinetic evolution models allow identification of the characteristic spectral signatures of the products and intermediates (Vernooij et al., 2018). Although the approaches have not yet been extensively explored to analyze the evolution of Raman spectroscopic signatures of cytological processes, initial applications to track the spectroscopic signatures of the *in vitro* action of the chemotherapeutic agent, doxorubicin, are promising (Perez-Guaita et al., 2020).

A number of technological variations on the basis of Raman spectroscopy have been developed to enhance the sensitivity of the technique. Surface Enhanced Raman Spectroscopy (SERS) was first observed in 1974 (Fleischmann et al., 1974; Jeanmaire and Van Duyne, 1977), and has been explored for the development of nanoparticle based analytical probes to monitor nanomaterials in a cellular environment (Kneipp et al., 2002, 2009). Incorporation of Raman reporters enables localization of the SERS probe within the subcellular environment, and such SERS nanosensors can be designed, for example to monitor pH changes in a cell at the different stages of the endocytic pathway (Kneipp et al., 2006, 2010; Wang et al., 2008). A number of studies have described novel SERS based probes for intracellular sensing and imaging (Kneipp et al., 2009), identification of biomarkers (Zhang et al., 2020) and to monitor intracellular delivery of single gold particles via 3D hollow nanoelectrodes (Huang et al., 2019). SERS has also been employed to probe cell surface receptors associated with cancer (Kong et al., 2012), in the isolation and non-invasive analysis of circulating cancer stem cells (Cho et al., 2018), and applications in cancer detection and tumor imaging have been extensively explored (Meola et al., 2018; Ravanshad et al., 2018).

The technique particularly exploits the enhanced local field of surface plasmon resonances in nanoparticles of, for example gold and silver. Only a small selection of nanoparticles can be exploited for such applications, however, and specific probes of specific cellular function are required, and therefore, in many cases, it is debatable whether the technique can really be considered label-free, although there have been notable examples of studies in which nanostructured gold/silver substrates have been used as SERS substrates for cellular investigation, in a label-free manner (Dipalo et al., 2015; La Rocca et al., 2015; Shalabaeva et al., 2017; Caprettini et al., 2018). The current manuscript highlights the applications of (unenhanced) Raman microspectroscopy itself to probe, in a label free manner, the cellular uptake, distribution and fate of nanoparticles themselves, applicable to a broad range of chemical compositions and morphologies, and to, using the same measurement protocol, probe the cellular response.

It is acknowledged that, unenhanced, the sensitivity of the technique is a potential limitation, resulting in prolonged analysis times for cellular imaging. Emerging techniques based on coherent Raman scattering, in the form of either coherent anti-Stokes Raman (CARS) or stimulated Raman scattering (SRS) promise to significantly enhance the sensitivity, and therefore reduce the sampling time, without compromising the label-free aspect of the technique. Although currently only commercially available in limited frequency ranges, full spectral coherent Raman microspectroscopic imaging has already been demonstrated (Camp et al., 2014), promising label free high content spectroscopic analysis of sub-cellular processes in real-time, and hitherto unrivaled visualization of cellular processes and function. Indeed, CARS has been employed, in conjunction with electron microscopy to explore cell-nanoparticle interactions at nanometer resolution (Saarinen et al., 2019), while SRS using polymer nanoparticles has been employed for multiplexed live-cell imaging (Hu et al., 2017).

## Conclusion

Nanomaterials, and in particular nanoparticles, have become an important element of the drive for the development of functional biomaterials, and although their potential in therapeutics and diagnostics is already well established, the “other side of the coin,” that of potential harmful effects to human health and the environment, is equally evident. The associated short and long term challenge, pertinent to both sides of the coin, relates to the ability to directly monitor and evaluate the interactions of these novel functional materials with living systems, and particularly cells. While conventional microscopic techniques and cytological staining and assays can provide a limited picture of the process, the technique of Raman microspectroscopy has been demonstrated to be capable of; confirming the intracellular localization of NPs in cells, monitoring the NP trafficking in subcellular vesicles and, monitoring NP degradation catabolization, identifying and tracking cellular response pathways, all in a single, label free, measurement protocol. The studies strongly suggest that the spectroscopic signatures are characteristic of key initiating and pathway events, although exploration of more sophisticated, dynamic multivariate data mining techniques is required to map out cellular response pathways. However, further larger scale studies are required to verify the transferability of the characteristic “spectralomic” signatures, for a range of different NP and cell types. Applications can readily be envisaged in areas such as toxicological screening, to guide regulatory processes, *in vitro* pre-clinical candidate drug screening to guide synthetic strategies, and ultimately potentially even patient screening for drug resistance/sensitivity, as the basis for companion diagnostics.

## Author Contributions

FB undertook the initial studies of PSNPs in cells. EE undertook the localization and toxicology studies of PSNPs, PS-NH_2_, and PAMAM in cells. CM and JM undertook the studies of MoS_2_ in THP-1 cells. HB is the senior responsible scientist and drafted the manuscript, with input from all other authors. All the authors contributed to the article and approved the submitted version.

## Conflict of Interest

The authors declare that the research was conducted in the absence of any commercial or financial relationships that could be construed as a potential conflict of interest. The handling editor declared a past co-authorship with several of the authors, HB, EE, and JM.
